# The path to « the Golden Age » for the treatment of metastatic renal cell carcinoma

**DOI:** 10.18632/oncotarget.25832

**Published:** 2018-08-03

**Authors:** Gilles Pagès

**Affiliations:** University of Nice Sophia Antipolis, Institute for Research on Cancer and Aging of Nice, CNRS UMR; INSERM U1081, Centre Antoine Lacassagne, France; Centre Scientifique de Monaco, Biomedical Department, 8 Quai Antoine Ier, Monaco, Principality of Monaco

**Keywords:** renal cell carcinoma, innovative treatments

Despite several improvements during the last decade, metastatic renal cell carcinoma (mRCC) remains incurable. However, mRCC represent a general paradigm of tumor vascularization. The molecular mechanism linked to the exacerbated angiogenesis in these tumors was considered as relatively simple: inactivation of the von Hippel Lindau (*VHL*) tumor suppressor gene in > 70% of sporadic cases. *VHL* inactivation is linked to constitutive expression of Hypoxia Inducible Factor 1 or 2 alpha (HIF- 1/2α) and therefore increased transcription of the Vascular Endothelial Growth Factor (VEGF) gene.

After diagnosis, the therapeutic strategy depends on the metastatic status: i) if no metastasis is detected by CT scan, treatment relies on partial or complete removal of the affected kidney then surveillance every six months; ii) in case of metastasis, two strategies are available; anti- VEGF monoclonal antibodies bevacizumab/Avastin in combination with interferon alpha induced a revolution in the field by increasing the time to progression of metastatic patients [1]; sunitinib, an inhibitor of several tyrosine kinase receptors involved in the mechanisms of angiogenesis including the VEGF (VEGFR1/2/3), PDGF, CSF1 receptors and c-Kit is now the reference treatment in the first line [2]. Other receptor tyrosine kinase or mTOR inhibitors are available for second- or third-line treatments including axitinib [3], pazopanib [4], cabozantinib [5], lenvatinib [6] and everolimus [7]. Immuno-therapies based on interleukin 2 or interferon alpha have also demonstrated efficacy before the development of anti-angiogenic drugs and recently new immunotherapies based on immune check-point inhibitors showed promising results for the first line treatment or at relapse on anti-angiogenics [8, 9].

The development of these different strategies is a “scholar case” and highlights renal cancers as one of the most active field for the development of new therapeutic tools. Hence, Hsieh *et al* described an evolution of renal cancers treatment, from the dark-age in the 1990s to the modern-age during the 2000s 2010s and we currently experiencing the beginning of the golden-age [10]. However, despite the development of more than 10 new treatments, the average overall survival has modestly increased. Thus, renal cancers remain incurable.

However, simple observations have to be emphasized according to patients’ specificity and treatments:

1. Despite equivalent clinico-pathological characteristics, tumors present a different response to the same treatment. The survival of 4543 patients treated with sunitinib ranged from few months to few years without satisfactory explanations [2]. This heterogeneity in response may rely on genetic heterogeneity of independent tumors [11].
2. Current treatments target tyrosine kinase receptors (VEGFR, PDGFR, FGFR, CSFR1 or c-KIT). More recently, targeting the c-MET pathway was considered relevant [5]. Inhibitors of programmed death receptor (PD1) or programmed death receptor ligand (PDL1) or cytotoxic T-lymphocyte-associated protein 4 (CTLA4) probably target the “emerged part of the iceberg” of proteins involved in immune-tolerance.

In accordance with these considerations, new strategies must be developed to chronicize or even to cure metastatic kidney cancers. The paper of Roelants *et al* proposes an elegant strategy based on the following rationale: i) several tyrosine kinase receptors are involved in exacerbated angiogenesis and growth of kidney cancers; ii) all tyrosine kinase receptors activate the PI3 Kinase and Src kinase. Hence, targeting paramount signaling pathways down-stream of tyrosine kinase receptors should be highly efficient and less submitted to tumor cell adaptation. The authors have focused on two drugs used in clinical trial; the GDC-0941 (PI3 Kinase inhibitor) and Saracatinib (Src Kinase inhibitor). Independently, these two drugs have a modest activity on different *in vitro* parameters of tumors cells aggressiveness. However, the authors observed a synergistic anti-tumor effect by combining both drugs. As described above, *VHL* inactivation happens in > 70% of renal cancers. However, the VHL wild-type tumors are more aggressive [12]. Roelants *et al* further showed that *VHL* negative cells re-expressing VHL are more sensitive to the GDC-0941/Saracatinib combination. Therefore their strategy might be relevant for highly aggressive tumors.

The *in vitro* results were confirmed on *ex vivo* explant cultures from renal tumor Patient-Derived Xenografts (PDXs) and experimental tumors in mice.

Together, the results of Roelants *et al* represent a real breakthrough in the field of treatment of renal cancers. Since the two tested drugs are currently in clinical trials for other pathologies, new phase I/II trials based on this relevant rational would deserve to be launched.
