# Correction: The path to « the Golden Age » for the treatment of metastatic renal cell carcinoma

**DOI:** 10.18632/oncotarget.26340

**Published:** 2018-11-06

**Authors:** Gilles Pagès

**Affiliations:** ^1^ University of Nice Sophia Antipolis, Institute for Research on Cancer and Aging of Nice, CNRS UMR; INSERM U1081, Centre Antoine Lacassagne, France; Centre Scientifique de Monaco, Biomedical Department, 8 Quai Antoine Ier, Monaco, Principality of Monaco

**This article has been corrected:** In the fifth paragraph of the article, the following sentence has been corrected to read: ‘Roelants *et al* further show that VHL negative cells re-expressing VHL are less sensitive to the GDC-0941/Saracatinib combination.’

In accordance with these considerations, new strategies must be developed to chronicize or even to cure metastatic kidney cancers. The paper of Roelants *et al* proposes an elegant strategy based on the following rationale: i) several tyrosine kinase receptors are involved in exacerbated angiogenesis and growth of kidney cancers; ii) all tyrosine kinase receptors activate the PI3 Kinase and Src kinase. Hence, targeting paramount signaling pathways down-stream of tyrosine kinase receptors should be highly efficient and less submitted to tumor cell adaptation. The authors have focused on two drugs used in clinical trial; the GDC-0941 (PI3 Kinase inhibitor) and Saracatinib (Src Kinase inhibitor). Independently, these two drugs have a modest activity on different *in vitro* parameters of tumors cells aggressiveness. However, the authors observed a synergistic anti-tumor effect by combining both drugs. As described above, *VHL* inactivation happens in > 70% of renal cancers. However, the VHL wild-type tumors are more aggressive [12]. Roelants *et al* further show that *VHL* negative cells re-expressing VHL are less sensitive to the GDC-0941/Saracatinib combination. Therefore their strategy might be relevant for highly aggressive tumors.

Original article: Oncotarget. 2018; 9:31564-31565. https://doi.org/10.18632/oncotarget.25832

